# Parallel And Divergent Morphological Adaptations Underlying The Evolution of Jumping Ability in Ants

**DOI:** 10.1093/iob/obad026

**Published:** 2023-07-25

**Authors:** L Aibekova, R A Keller, J Katzke, D M Allman, F Hita-Garcia, D Labonte, A Narendra, E P Economo

**Affiliations:** Biodiversity and Biocomplexity Unit, Okinawa Institute of Science and Technology Graduate University, 1919-1 Tancha, Onna-son, Okinawa 904-0495, Japan; Museu Nacional de Historia Natural e da Ciência & Centre for Ecology, Evolution and Environmental Changes & CHANGE - Global Change and Sustainability Institute, Universidade de Lisboa, Lisbon, Portugal; Biodiversity and Biocomplexity Unit, Okinawa Institute of Science and Technology Graduate University, 1919-1 Tancha, Onna-son, Okinawa 904-0495, Japan; Ecological Neuroscience Group, School of Natural Sciences, Macquarie University, Sydney, NSW 2109, Australia; Biodiversity and Biocomplexity Unit, Okinawa Institute of Science and Technology Graduate University, 1919-1 Tancha, Onna-son, Okinawa 904-0495, Japan; Department of Bioengineering, Imperial College London, London SW7 2AZ, UK; Ecological Neuroscience Group, School of Natural Sciences, Macquarie University, Sydney, NSW 2109, Australia; Biodiversity and Biocomplexity Unit, Okinawa Institute of Science and Technology Graduate University, 1919-1 Tancha, Onna-son, Okinawa 904-0495, Japan

## Abstract

Jumping is a rapid locomotory mode widespread in terrestrial organisms. However, it is a rare specialization in ants. Forward jumping has been reported within four distantly related ant genera: *Gigantiops, Harpegnathos, Myrmecia*, and *Odontomachus*. The temporal engagement of legs/body parts during jump, however, varies across these genera. It is unknown what morphological adaptations underlie such behaviors and whether jumping in ants is solely driven directly by muscle contraction or additionally relies on elastic recoil mechanism. We investigated the morphological adaptations for jumping behavior by comparing differences in the locomotory musculature between jumping and non-jumping relatives using X-ray micro-CT and 3D morphometrics. We found that the size-specific volumes of the trochanter depressor muscle (*scm6*) of the middle and hind legs are 3–5 times larger in jumping ants, and that one coxal remotor muscle (*scm2*) is reduced in volume in the middle and/or hind legs. Notably, the enlargement in the volume of other muscle groups is directly linked to the legs or body parts engaged during the jump. Furthermore, a direct comparison of the muscle architecture revealed two significant differences between jumping vs. non-jumping ants: First, the relative Physiological Cross-Sectional Area (PCSA) of the trochanter depressor muscles of all three legs were larger in jumping ants, except in the front legs of *Odontomachus rixosus* and *Myrmecia nigrocincta*; second, the relative muscle fiber length was shorter in jumping ants compared to non-jumping counterparts, except in the front legs of *O. rixosus* and *M. nigrocincta*. These results suggest that the difference in relative muscle volume in jumping ants is largely invested in the area (PCSA), and not in fiber length. There was no clear difference in the pennation angle between jumping and non-jumping ants. Additionally, we report that the hind leg length relative to body length was longer in jumping ants. Based on direct comparison of the observed vs. possible work and power output during jumps, we surmise that direct muscle contractions suffice to explain jumping performance in three species, except for *O. rixosus*, where the lack of data on jumping performance prevents us from drawing definitive conclusions for this particular species. We suggest that increased investment in jumping-relevant musculature is a primary morphological adaptation that separates jumping from non-jumping ants. These results elucidate the common and idiosyncratic morphological changes underlying this rare adaptation in ants.

まとぅみ (Okinawan language—Uchinaaguchi)

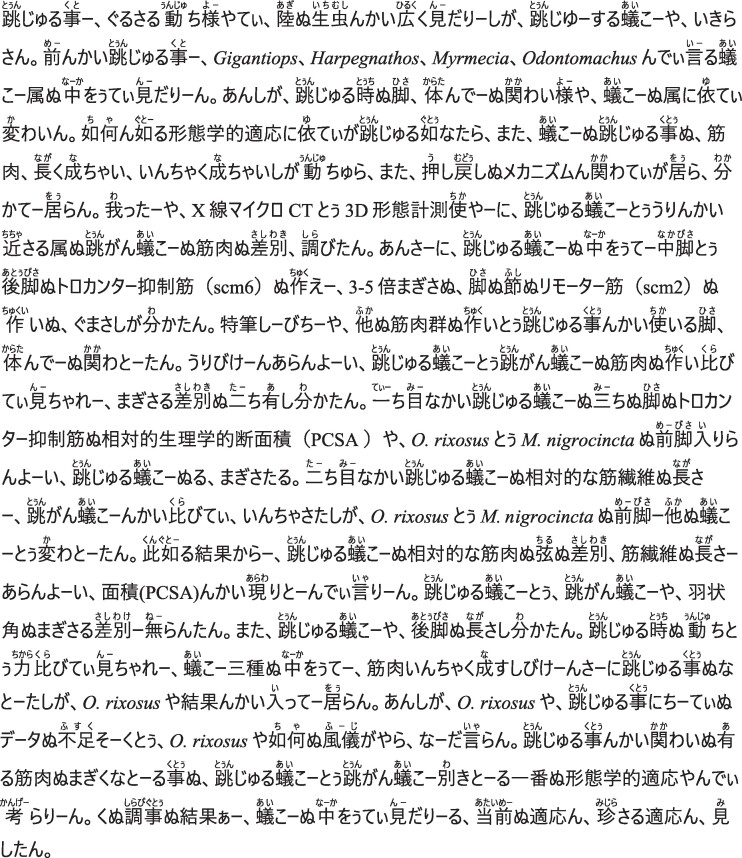

要旨 (Japanese)

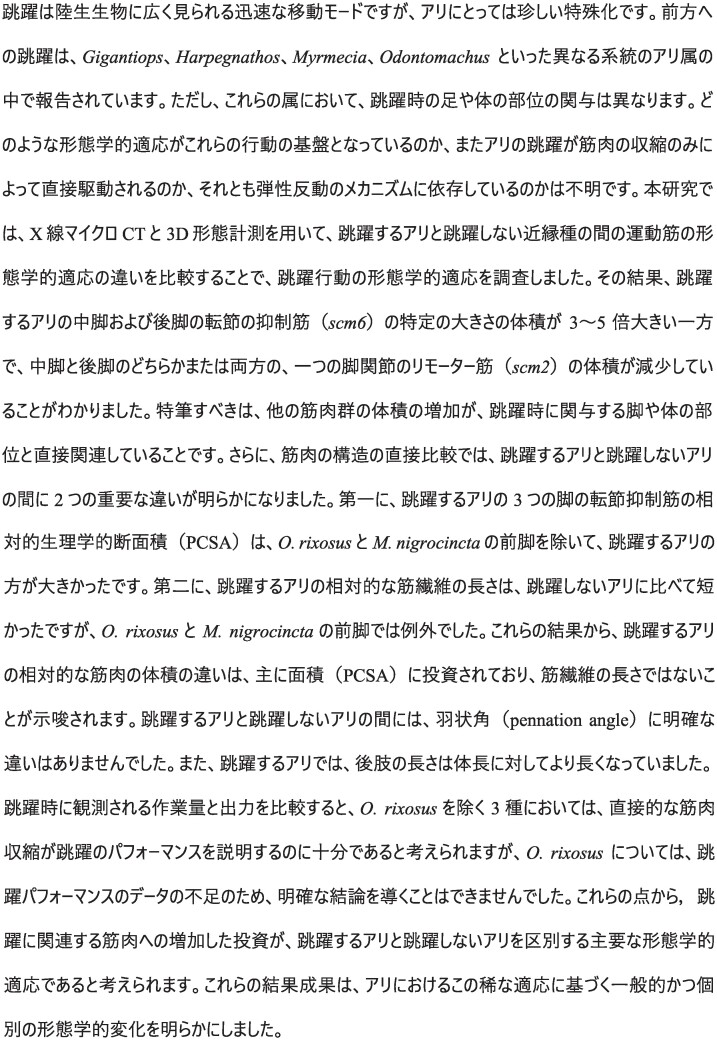

РЕЗЮМЕ (Kazakh)

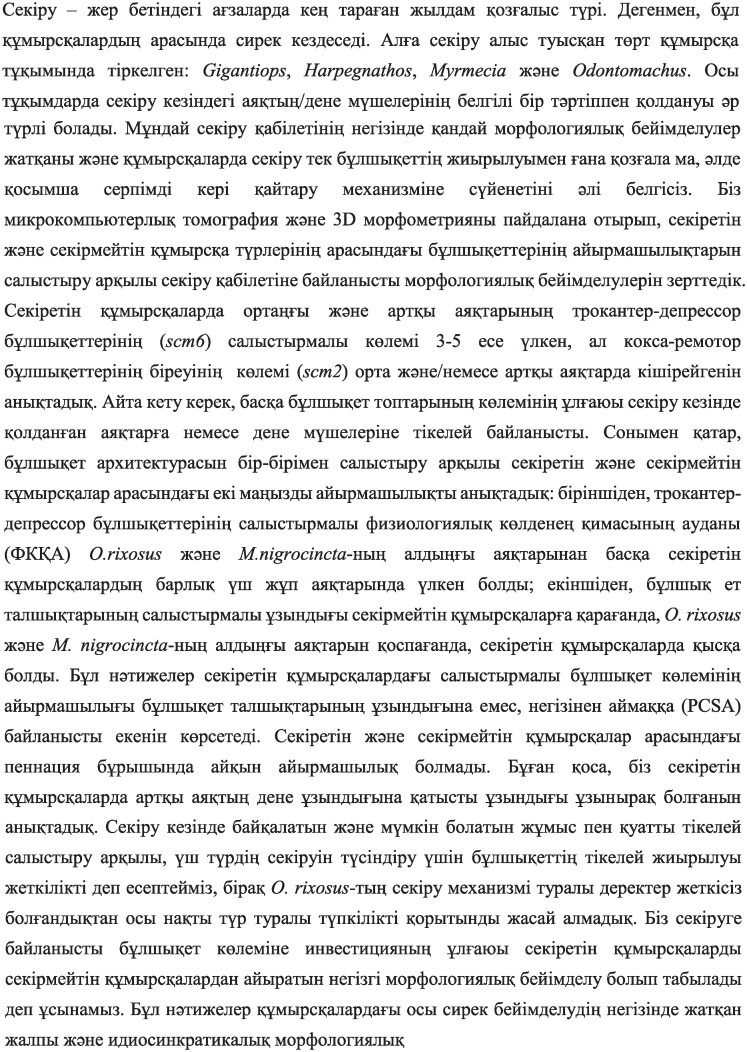

ZUSAMMENFASSUNG (German)

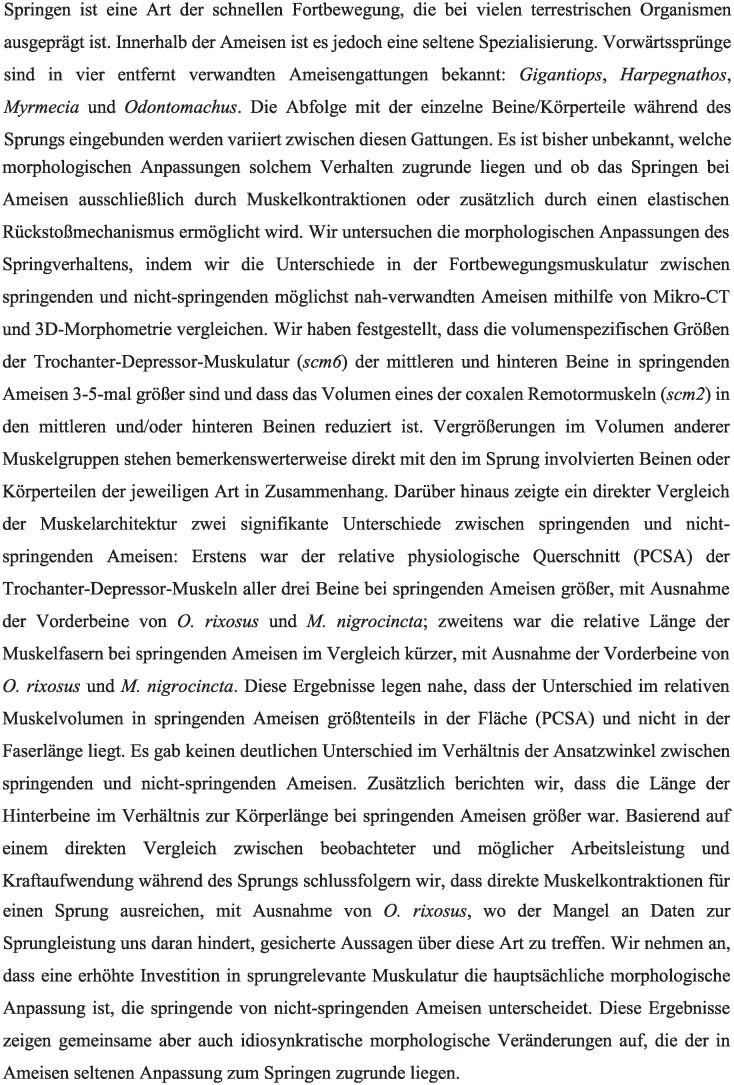

## Introduction

Understanding the coevolution between morphology and behavior is one of the central challenges in evolutionary biology. Changes in environment and competition for resources can trigger new innovations in behavior, which in turn lead to morphological and physiological adaptations (Wcislo 1989). Similar desired functionality can either result in convergent evolution of morphology, especially when a limited range of forms is readily accessible to evolution (McGhee 2011), or manifest itself in many-to-one mapping (Wainwright et al. 2005; Moen 2019), where the same functional trait is achieved by a diversity of morphological “design solutions.”

The evolution of jumping behaviors provides an excellent opportunity to study the relationship between function and morphology, because it often involves a combination of structural transformations, e.g., enlargement of muscular volumes and associated tendons, expansion of skeletal elements for larger attachment areas, and adaptations for elastic energy storage (Gorb 2004; Ogawa and Yoshizawa 2017). Furthermore, previous studies on insects have revealed a variety of distinct morphological designs that promote jumping. For example, locusts (Bennet Clark 1975), click beetles (Bolmin et al. 2021), and froghoppers (Burrows 2006) use catapult mechanisms to jump; other insects, such as mantises (Sutton et al. 2016), bush crickets (Burrows and Morris 2003), and moths (Burrows and Dorosenko 2015), in turn, rely on direct muscle actuation without additional contributions from elastic elements.

**Fig. 1:**
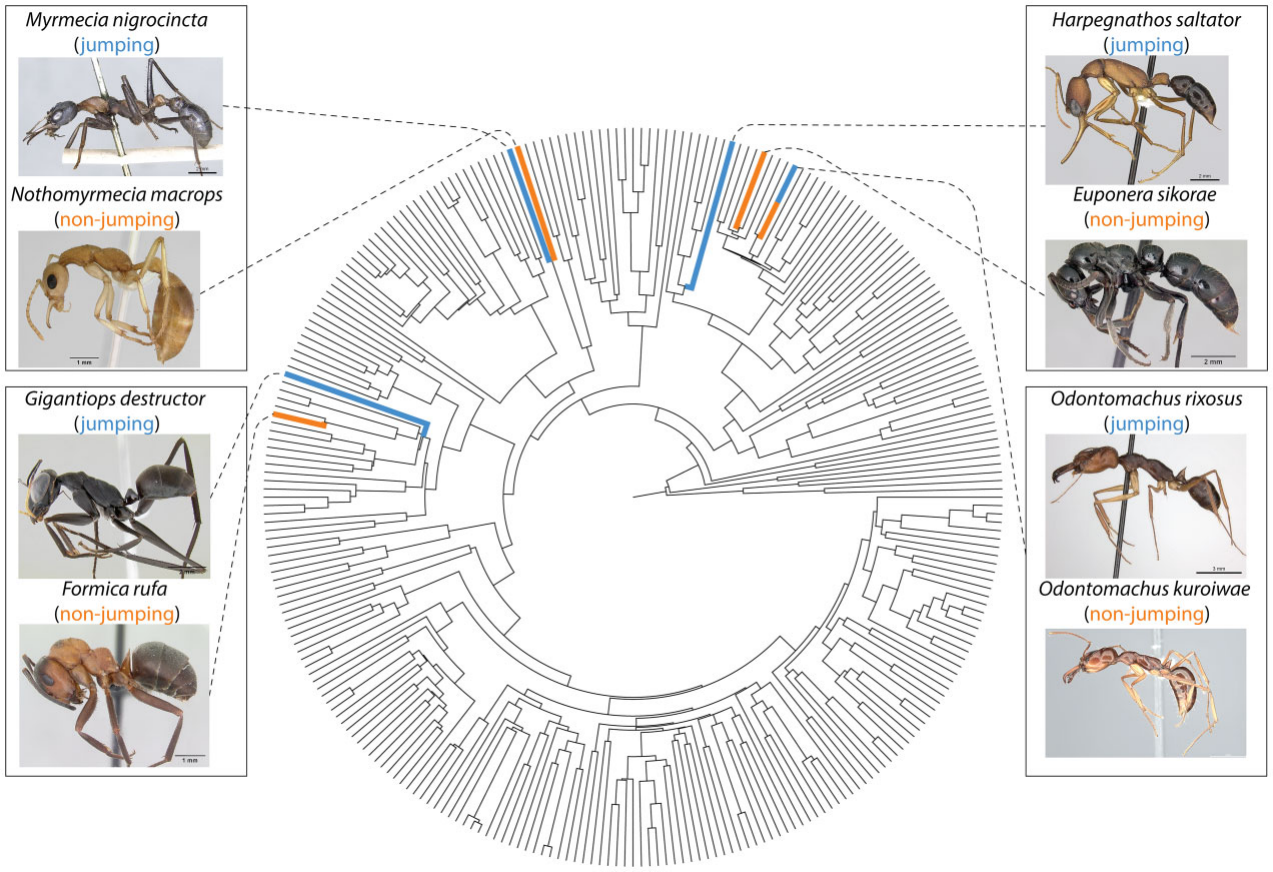
Phylogenetic relationship of the ant species in this study. The phylogenetic tree is from Economo et al. (2018) (the tree was trimmed to include one species per genus). Ant images from Antweb (antweb.org). (*E. sikorae*: CASENT0497202, *F. rufa*: CASENT0173862, *G. destructor*: CASENT0106169, *H. saltator*: CASENT0260424, *M. nigrocincta*: CASENT0902805, *N. macrops*: CASENT0172003, *O. rixosus*: CASENT0217544, *O. kuroiwae*: CASENT0741360.)

Despite the astounding diversity of ants, the ability to jump is rare; it has been reported in only six genera (Wheeler 1922; Ali et al. 1992; Baroni Urbani et al. 1994; Tautz et al. 1994; Sorger 2015). Jumping ants can be divided into two broad groups: prosalient, that is forward jumping using legs; and retrosalient, i.e., backward jumping using mandibles (Wheeler 1922; Patek et al. 2006). Retrosalience is observed in trap-jaw ants such as *Odontomachus* Latrielle 1804, *Strumigenys* Smith F. 1860, and *Anochetus* Mayr 1861, which use large muscles in their head to store elastic energy in both tendons and the head capsule (Sutton et al. 2022), which is then rapidly released, resulting in an impact between mandibles and the ground, and upward propulsion. Prosalient ants, on the other hand, use their legs to power a directed forward jump. Prosalience has evolved in four distantly related ant genera (see Fig 1): *Harpegnathos* and *Odontomachus* (Ponerinae), *Gigantiops* (Formicinae), and *Myrmecia* (Myrmeciinae) (Ali et al. 1992; Baroni Urbani et al. 1994; Tautz et al. 1994; Sorger 2015).

Most studies on prosalience in ants have focused on the kinematics and energetics of the movements (Ali et al. 1992; Baroni Urbani et al. 1994; Tautz et al. 1994; Ye et al. 2020). In contrast, there has been little fundamental research on jumping abilities in ants from a functional morphology perspective. Although we know how fast these ants jump, we do not yet understand what the adaptations to this function are, and how they differ from non-jumping relatives. Studying the gross anatomy will help us identify the groups of muscles that are essential to facilitate jumps and how they are modified.

All prosalient ants rely on middle and hind legs to jump (Tautz et al. 1994). According to Burrows (2011), there are at least two reasons for using four legs as opposed to a two-legged jump: First, more legs presumably result in a larger net ground reaction force, and so in improved jump performance. Second, it may help to control rotation of the body, so that most muscle work flows into kinetic energy of the Center of Mass (CoM) instead. It has been suggested that the front leg is of minor importance in jumping, and instead acts as a support for maintaining static equilibrium as in case of walking or running (Full and Tu 1991; Zollikofer 1994). Although all forward jumping ants universally use the middle and hind legs to jump, the engagement of different body parts during the jump may vary. For example, *Harpegnathos saltator* first uses the hind legs to move the body forward, and only later engages the middle legs to provide a final push (Tautz et al. 1994). However, Baroni Urbani et al. (1994) have shown that the muscle activities in the ipsilateral (same side) middle and hindlegs are synchronous, and thus have concluded that both legs extend in unison. *Gigantiops destructor*, in addition to using both middle and hind legs for propulsion, show a conspicuous and consistent movement of the metasoma (Tautz et al. 1994; Ye et al. 2020), and although the functional significance of this movement is unclear, its consistency suggests it is important. Lastly, *Myrmecia nigrocincta* are thought to use both the middle and hind legs simultaneously to propel the jump (Tautz et al. 1994). As the forward jumping ability of *Odontomachus rixosus* was only recently discovered (Sorger 2015), the temporal engagement of legs during its jump has not yet been studied.

The involvement of different body parts during jumping may be related to different strategies that would minimize net torque. The position of their CoM in this case is important, since the center of rotation is often located in the CoM. This is supported by the observation that the CoM location differs between jumping ant species (Tautz et al. 1994). The impulsive forces generated by the feet in ground contact may be converted into rotational and/or translational kinetic energy (Goode and Sutton 2023). Rotational kinetic energy may be a particular problem for small animals, as their mass moment of inertia is relatively smaller. The split in rotational vs. translational kinetic energy will be determined by the net torque. Zero rotation will only result if the net torque is zero. In *H. saltator*, the CoM is located in front of the middle leg (Tautz et al. 1994). The use of the middle legs to give the final propulsion could minimize the net torque. The CoM in *G. destructor* is located posterior to the legs, at the insertion of the petiole to the mesosoma (Tautz et al. 1994). The rotation of the metasoma could be to shift the CoM dynamically to minimize net torques generated by the legs. A similar behavior is observed in juvenile wingless mantises, which rotate their abdomen during their jump to adjust the mass moment of inertia (Burrows et al. 2015). In *M. nigrocincta*, the CoM is located between the middle and hind legs (Tautz et al. 1994), and perhaps the use of both legs would reduce the total net torque. As such, the differences in the jumping techniques in these ants could be due to the differences in the position of the CoM (Tautz et al. 1994).

There are two size-specific mechanisms used by jumping animals: one powered by direct muscle contraction and one relying on spring-actuated jumps (Sutton et al. 2019). In muscle-actuated jumps, jumping performance is constrained by the physiological properties of the muscle, such as its work density and intrinsic shortening speed. Thus, a closer look at the muscle architecture and volume may elucidate the muscular design for optimal force production.

As animals get smaller in size, the amount of mechanical energy that can be generated is instead limited by the force–velocity properties of the muscle, and it can become beneficial to rely on specialized morphological adaptations that improve performance through rapid recoil of elastic structures (Bobbert 2013; Sutton et al. 2019). Often, jump enhancement by elastic energy storage involves latch mechanisms: Muscles contract slowly to store strain energy in specialized cuticular structures, and this energy is subsequently rapidly released to power the jump (Bennet Clark 1975; Gronenberg 1996; Burrows 2003; Longo et al. 2019); although such “power amplification” overcomes force–velocity limitations, it nevertheless ultimately depends on the mechanical work output of muscle. Elastic energy stores in insects are diverse. Click beetles store energy in a specialized structure located in their thorax ventrally between front and middle legs (Bolmin et al. 2021). Locusts use hind femur muscles to load strain energy into the semi-lunar process (Bennet Clark 1975). Another energy storage site is a locking mechanism found in the femoro-tibial joint (Földvári et al. 2019). So far, it is unknown whether the evolution of forward jumping in ants involves elastic energy storage mechanisms.

The aim of this study is to investigate the morphological adaptations that underlie jumping ability in ants by comparing relative muscle volume, muscle architecture, and leg lengths of distantly related forward-jumping ants with their non-jumping relatives. We ask, (i) whether jumping behavior is associated with morphological adaptations to the locomotory system, and whether these include enlargement of muscles and/or adaptations for power amplification; and (ii) whether those morphological adaptations are consistent across distantly related lineages (i.e., 100my divergence), indicating convergent evolution, or if different biomechanical solutions underlie the independent evolution of jumping abilities. The results will inform our understanding of jumping in ants and, more generally, how the interplay between morphology and behavior affect diversification.

## Materials and methods

### Material

We selected one worker specimen preserved in ethanol (70–99%) from each genus for which jumping behavior has been previously documented, to carry out detailed computed tomography (CT) scanning and 3D morphometry: *Harpegnathos saltator* Jerdon 1851 (unique specimen identifier: CASENT0764679), *G. destructor* Fabricius 1804 (CASENT0709414), *O. rixosus* Smith F. 1957 (CASENT9741319) and *M. nigrocincta* Smith F. 1858 (CASENT0741302). As a control, we imaged a small set of non-jumping ant species: *Euponera sikorae* Forel 1891 (CASENT0709898)*, Formica rufa* Linnaeus 1761 (CASENT0741323), *Odontomachus kuroiwae* Matsumura 1912 (CASENT0741313) and *Nothomyrmecia macrops* Clark 1934 (CASENT0795539). An effort was made to select comparison species as closely related as possible to the jumping species considering availability of preserved specimens. *Harpegnathos* has no close relatives, and all species in the genus are known to jump to our best knowledge. Thus, any non-jumping comparator is necessarily phylogenetically distant. In some phylogenies (e.g., Economo et al. 2018), there are a few genera that are marginally closer than *Euponera*, but in other trees *Harpegnathos* is sister to the rest of the *Ponerinae*. For specimen preparation, see Katzke et al. (2022). The length of the leg segments was measured three times within the same specimen (in the right and/or left legs). For the specimens for which no information on the body mass was available, the whole body was scanned, and body volume was used as proxy for body mass via assuming a uniform density of $1040\ {\rm{kg}}/{{\rm{m}}}^3$: *E. sikorae* (CASENT0741359); *F. rufa* (ANTSCAN, CASENT0709272); *G. destructor* (CASENT0744574); *N. macrops* (CASENT0741364).

### Micro-CT scanning and 3D-reconstruction

Micro-CT scans were generated with a Zeiss Xradia 510 Versa 3D X-ray microscope operated with the Zeiss Scout-and-Scan Control System software (version 14.0.14829.38124) at the Okinawa Institute of Science and Technology Graduate University, Japan. Scans were conducted with a 40 kV (75 μA)/3 W beam strength under a 4× magnification. Voxel size and exposure time depended on specimen size (Supplementary Table S1). As the mesosoma of ants exceeds the field-of-view of the camera at high magnification, vertical stitching of serial scans was used. 3D reconstructions of the resulting scan projection data were done with the Zeiss Scout-and-Scan Control System Reconstructor (version 14.0.14829.38124) and saved in txm file format. Postprocessing of txm raw data was done with Amira 2019.2 (Visage Imaging GmbH, Berlin, Germany) to segment individual structures into discrete tissue volumes. The segmented voxels were then exported with the plugin script “multiExport” (Engelkes et al. 2018) in Amira 2019.2 as 2D TIFF image stacks. VG-Studio 3.4 (Volume Graphics GmbH, Heidelberg, Germany) was used to create volume renders from the TIFF image series. Muscle architecture was reconstructed with Amira 2019.2 XTracing extension, following the workflow presented in Katzke et al.(2022). The accuracy of the tracing algorithm in Katzke et al. (2022) was 92% for fiber length estimation and 100% for the pennation angle estimation. Muscle identity and nomenclature follows Aibekova et al. (2022). Muscles most relevant in the movement of the legs were segmented (Ipcm2, Iscm4, I-, II-, IIIscm1, II-, IIIscm2, I-, II-, IIIscm3, Ipcm8, II-, IIIscm6, Ipcm4, II-, IIIpcm3_4, I-, II-, IIIctm1, I-, II-, IIIctm2, I-, II-, IIIctm3); in addition, large muscles, including the indirect muscle of the head (Idvm5), the levator (IA1), and one of the rotators (IA2) of abdomen were segmented for control. We want to mention one caveat related to preparation technique. It is possible that there may be different degrees of muscle shrinkage due to the preservation in high ethanol concentrations (70–99%). This could in principle cause spurious differences across species, as the level of contraction may vary among species, however we have no evidence this was an issue in this case, and it is unlikely to affect the broad differences identified in this study.

### Data analysis

Our study design was based on comparing related pairs of jumping and non-jumping species, which should account for phylogenetic signal. However, due to the low throughput of recovering detailed scan and segmentation data for each specimen, and the low number of known independent evolutions of forward jumping (4), we could not perform formal statistical comparative analyses with large numbers of jumping and non-jumping species. Thus, although we quantify morphological differences, our study was mainly performed by comparing values directly, a common limitation of studies of phenotypes without large numbers of independent evolutions. Given the low sample sizes, this exploratory approach can characterize broad and consistent differences but not subtle changes that require large sample sizes. Measurement error and intraspecific variation were assessed however, to ensure our characterization of interspecific differences are not obscured by other sources of variation. For this, we scanned and segmented four *Myrmecia croslandi* specimens from two collection events: *M. croslandi* rep 0 (CASENT0741321), *M. croslandi* rep 1 (CASENT0741324), *M. croslandi* rep 2 (CASENT0741305), and *M. croslandi* rep 3 (CASENT0741308). Rep 0 and rep 1 are from an old collection stored in 90% ethanol, Rep 2 and 3 are from the recent collection, stored in 70% ethanol.

In addition to comparing values of morphological parameters (e.g., volumes, fiber lengths) directly, principal component analysis (PCA) was conducted on centered and scaled absolute muscle and thorax volumes to quantify variation in multidimensional space.

### Scaling

To compare the morphology across ants of different body sizes, the muscle volume, PCSA, and fiber length were normalized: ${V}_{muscle} \propto V_{thorax}^1$, $PCSA \propto V_{muscle}^{0.66}$, and ${L}_{fiber} \propto V_{muscle}^{0.33}$. In the absence of detailed information on the force-length properties of the involved muscles (Püffel et al. 2023), we define PCSA as $\frac{{Volume}}{{length}}$. Information on the body mass was lacking for some ants. The data on the volumes of different body parts (head, thorax, petiole, and gaster) of ants, that were calculated from the linear measurements from a single representative species from 231 genera, were available from Anderson, Rivera, and Suarez (2020). We used these data to confirm that thorax volume scales isometrically with total body volume (slope of 1.021 (95% CI: 1.003–1.039), Supplementary Fig. S1). Thus, we used the volume of the thorax as a proxy for body mass.

### Jumping kinematics in *M. nigrocincta*

Workers of *M. nigrocincta* exhibit limited variation in body size (Sheehan et al. 2018), and we established kinematics here from one individual that carried out 3 consecutive jumps. *Myrmecia nigrocincta* are visually oriented ants, and we found that they jump in a lab setting to a vertical feature. Hence, we provided them with a horizontal jumping platform and a landing platform with a vertically placed piece of bark that was 3.8 cm away. We filmed the jumps at 6000 frames per second with an inter-frame interval of 0.166 ms with a resolution of 1920 × 1080 pixels. We filmed the jumps using a Phantom T1340 high-speed camera, 105 mm f2.8 Sigma macro lens coupled with custom-built LED lights. Immediately after filming, we weighed the ant on a scale and preserved it in 70% ethanol. We identified the CoM of this individual by balancing the ant on an insect pin and found this to be between the coxae of the mid and hind legs. We tracked the CoM in DLTdv8, a Matlab based application for digitizing video (Hedrick 2008). From this, we determined the take-off time (duration between the first movement of the propulsive leg and the first instance when no legs touched the ground, ms), take-off velocity (displacement of CoM over time, m/s), and acceleration (m/s^2^). A comparative analysis of jumping kinematics in *Myrmecia* is ongoing, where we are investigating both phylogenetic differences and the effect of size.

### Data availability

Supplementary Table S1 contains all the specimen data used in this study. Each specimen can be traced by a unique specimen identifier included in the preservation vial. The original µCT scans are available in DICOM format as well as the raw data on the muscle volume, PCSA, pennation angle and fiber length are available at the Dryad Digital Repository (https://doi.org/10.5061/dryad.v41ns1s15). In addition, we have provided freely accessible 3D models of the mesosoma segmentations of all species studied on Sketchfab (https://skfb.ly/oBzMA).

## Results

### Difference in relative volume of muscles involved in jumping

**Fig. 2:**
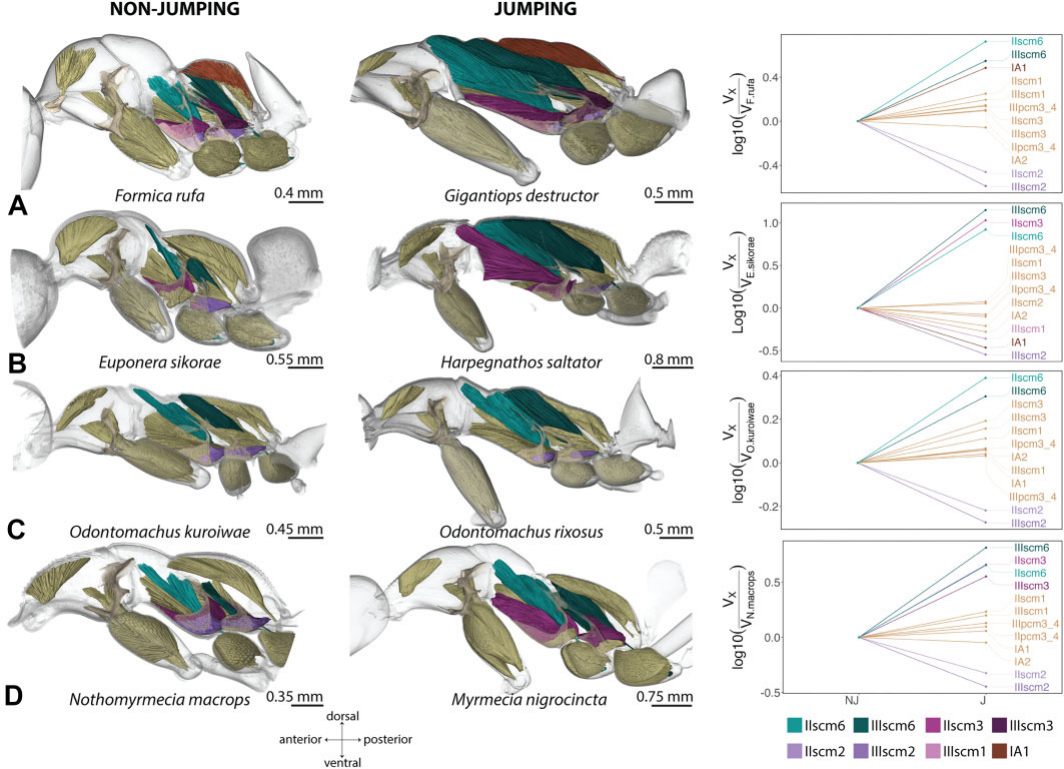
Comparison of the muscle volumes of the leg (coxal and trochanter) muscles. **A**. *F. rufa* and *G. destructor* pair; **B**. *E. sikorae* and *H. saltator* pair; **C**. *O. kuroiwae* and *O. rixosus* pair; and **D**. *N. macrops* and *M. nigrocincta* pair. The line graph on the right side shows the change in the relative volume of the muscles in jumping ant. Muscles which changed the most are colored. The trochanter depressor (scm6) muscles are enlarged in all jumping ants in both middle and hindlegs, while one of the coxal remotor (scm2) muscles are reduced in middle and/or hindleg. *Gigantiops destructor* in addition to using the middle and hind legs synchronously, rotates its metasoma to jump; *H. saltator* first uses its hind legs to move the body forward, then the middle legs to give final propulsion; *M. nigrocincta* uses the middle and hind legs synchronously to jump.

To understand the mechanisms underlying the jumping ability of ants, we compared the normalized muscle size and architecture of several key muscle groups between four jumping ant genera and four closely related non-jumping ant genera. All four jumping ant genera showed changes in size-specific muscle size and architecture of several key muscle groups. Within the meso- and metathorax of jumping species, the trochanter depressor muscles (IIscm6–*M. mesofurca-trochanteralis*; IIIscm6–*M. metafurca-trochanteralis*) occupy a large portion of the cavity space (Fig. 2 and Suppl. Fig. S2). In *G. destructor* the relative volume of the trochanter depressor muscle is 5 times larger in the middle legs and 3.5 times in the hind legs (Model 1) compared to *F. rufa* (Aibekova et al. 2022, Model 3); in *H. saltator* it is 8 times larger in the middle and 14 times larger in the hind legs (Model 2) compared to *E. sikorae* (Model 3); in *M. nigrocincta* it is 4.5 and 6.5 times larger in the middle and hind legs (Model 4) compared to *N. macrops* (Model 5); and in *O. rixosus* it is 2.5 and 2 times larger in the middle and hind legs (Model 6) compared to *O. kuroiwae* (Model 7). The trochanter depressor muscle originates at the furcal arms, the anterodorsal pleural region, and the notum of the meso- and metathorax; it inserts on the trochanter via a long tendon. The homologous muscle in the front leg (Ipcm8) is of similar relative volume in *H. saltator* and *O. rixosus* compared to their non-jumping counterpart, and in *G. destructor* and *M. nigrocincta* it is two times larger compared to their non-jumping counterpart (Supplementary Fig. S3).

Another muscle that is enlarged in relative volume in jumping ants compared to non-jumping ants is one of the remotors of the coxa (IIscm3–*M. mesofurca-coxalis medialis*; IIIscm3–*M. metafurca-coxalis medialis*). In *O. rixosus*, scm3 of both middle and hind legs are around 1.5 times larger than in *O. kuroiwae*. In *H. saltator*, scm3 of the middle legs is 10 times larger than in *E. sikorae*, but in the hind legs it is slightly smaller (0.8 times). In addition, the levator muscle of petiole (IA1) is 3 times larger in *G. destructor* compared to *F. rufa*, but the relative volume of IA1 in other pairs is similar.

IIscm2 (IIscm2–*M. mesofurca-coxalis posterior*) and IIIscm2 (IIIscm2–*M. metafurca-coxalis posterior*), which are remotors of the coxa (along with scm3 muscles) are reduced in jumping ants (0.2–0.61 times smaller), compared to the non-jumping pairs. 0.35 and 0.26 times smaller in *G. destructor*; 0.62 and 0.28 times smaller in *H. saltator*; 0.47 and 0.36 times smaller in *M. nigrocincta*, and 0.61 and 0.53 times smaller in the middle and the hind legs of *O. rixosus* compared to their non-jumping counterparts.

**Fig. 3:**
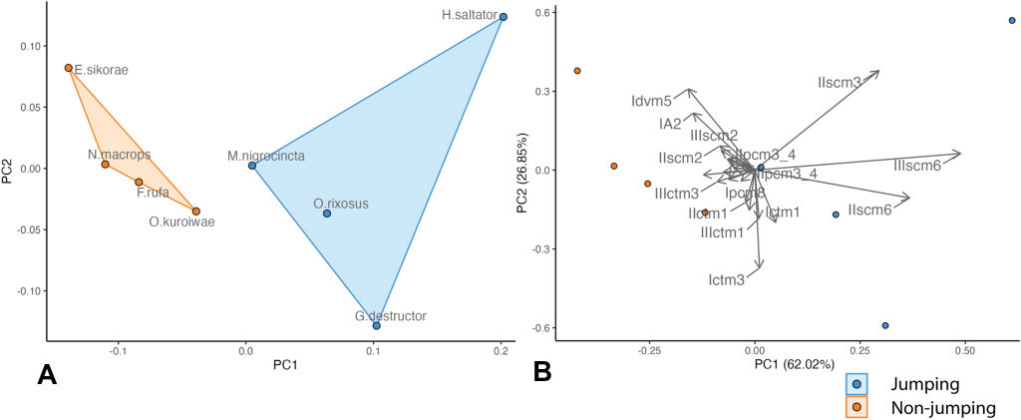
Principal Component Analysis (PCA) of the absolute volumes of muscles and the thorax in ants. **A.** Score plot of the principal components 1 and 2 (PC1 and PC2). **B.** Loadings plot of the PC1 and PC2. The blue dots represent jumping ants and orange represents non-jumping ants. PC1 axis represents 62.02% of the variation, separates jumping and non-jumping ants.

Principal component analysis on the absolute volumes of the muscle and thorax, has shown the separation of jumping and non-jumping ant species on Principal component 1 (PC1) axis (Fig. 3); it explained 62.02% of the variation in the sample and PC 2 explained 26.85% of variation in the sample. Most of the variation on PC1 axis come from three muscles: trochanter depressor muscles of the middle and hind legs (IIscm6 and IIIscm6) and coxal remotor muscle of the middle leg (IIscm3).

### Muscle architecture

#### Physiological cross-sectional area

**Fig. 4:**
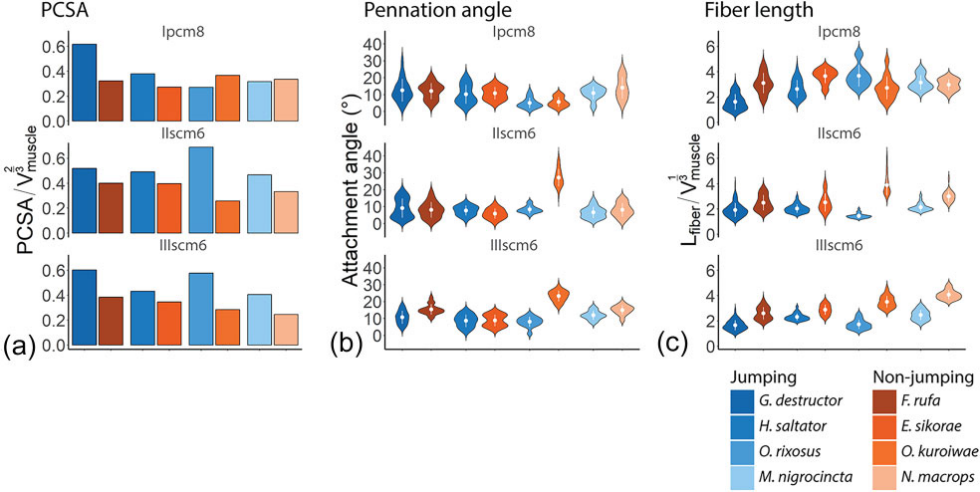
Muscle architecture estimations following fiber tracing of trochanter depressor muscle in the fore (Ipcm8), middle (IIscm6), and hind (IIIscm6) legs. **A**. Effective Physiological Cross-Sectional Area (_EFF_PCSA). To normalize for ant size, we divided the _EFF_PCSA by the approximation of the Surface Area (SA) of the thorax; **B**. Violin plot of the distribution of attachment (pennation) angles, the white dot indicates the mean value, and the white line indicates the s.d.; **C**. Violin plot of the distribution of muscle fiber length, the white dot indicates the mean value, and the white line indicates the s.d.

The trochanter depressor muscles of the front (Ipcm8), the middle (IIscm6), and hind legs (IIIscm6) of jumping ants have larger relative PCSA values (normalized to $V_{muscle}^{0.66}$) compared to non-jumping counterparts (Fig. 4A and Supplementary Table S2), except in the trochanter depressor muscle of the front legs (Ipcm8) of *O. rixosus* and *M. nigrocincta*, where the relative PCSA of was smaller. In *G. destructor*, the relative PCSA of the trochanter depressor muscle of the front, middle, and hind leg was 1.91, 1.30, and 1.57 times larger, respectively, compared to *F. rufa*. Similarly, in *H. saltator*, the relative PCSA of the trochanter depressor muscle of the front, middle, and hind legs were 1.39, 1.24, and 1.25 times larger compared to *E. sikorae*. In *O. rixosus*, in turn, the relative PCSA of the trochanter depressor muscle the front, middle, and hind legs were 0.74, 2.67, and 2.03 times larger compared non-jumping *O. kuroiwae*. In *M. nigrocincta*, the relative PCSA of the trochanter depressor muscle of the front, middle, and hind legs were 0.94, 1.40, and 1.65 times larger compared to *N. macrops*.

#### Pennation angle

The mean pennation angle (±s.d., *N*—number of muscle fibers) in the trochanter depressor muscle in *Gigantiops* and *Formica* pair was similar in the front (13°±7, *N* = 77 and 12°±5, *N* = 45 accordingly) and middle legs (9°±6, *N* = 210 and 8°±5, *N* = 31 accordingly). In the hind legs, in turn, it was smaller in *G. destructor* (11°±4, *N* = 72), compared to *F. rufa* (15°±3, *N* = 39) (Fig. 4B and Supplementary Table S2). Similarly, in *Harpegnathos* and *Euponera* pair, the mean pennation angle in the front (10°±6, *N* = 29 and 11°±4, *N* = 17 respectively) and hind legs (9°±4, *N* = 91 and 9°±3, *N* = 18, respectively) was similar, while in the middle legs it was slightly larger in *H. saltator* (8°±3, *N* = 97), compared to *E. sikorae* (6°±3, *N* = 19). The mean pennation angle of the trochanter depressor muscle was smaller in all three legs of *M. nigrocincta* (11°±4, *N* = 16 in the front, 7°±3, *N* = 46 in the middle, and 12°±3, *N* = 23 in the hind legs) compared to *N. macrops* (14°±6, *N* = 16 in the front, 8°±4, *N* = 26 in the middle, and 15°±3, *N* = 11 in the hind legs). In *Odontomachus* pairs, the mean pennation angle in the middle and hind legs was larger in non-jumping *O. kuroiwae* (27°±5, *N* = 28 in the middle and 23°±3, *N* = 29 in the hind legs), compared to jumping *O. rixosus* (8°±2, *N* = 17 in the middle and 8°±3, *N* = 39 in the hind legs), while in the front legs it was similar (6°±3, *N* = 14 and 5°±3, *N* = 21 accordingly).

#### Muscle fiber length

The normalized mean fiber length (±s.d., *N*—number of muscle fibers) of the trochanter depressor muscle (relative to $V_{muscle}^{0.33}$) is shorter in jumping ants compared to their non-jumping counterparts in all three legs, except in the front leg of *O. rixosus* and *M. nigrocincta* (Fig. 4C and Supplementary Table S2). In *G. destructor* the relative mean length of fibers was 1.62 (±0.61, *N* = 77), 1.92 (±0.55, *N* = 210), and 1.66 (±0.40, *N* = 72) in front, middle, and hind legs, respectively, shorter than those of *F. rufa* (3.1 ± 0.8, *N* = 45, 2.49 ± 0.6, *N* = 31, and 2.6 ± 0.49, *N* = 39). In *H. saltator*, the relative length of muscle fibers 2.63 (±0.71, *N* = 29), 2.03 (±0.34, *N* = 97), and 2.32 (±0.28, *N* = 91) in the front, middle, and hind legs, while in *E. sikorae* it was 3.66 (±0.57, *N* = 17), 2.52 (±0.71, *N* = 19), and 2.89 (±0.4, *N* = 18) accordingly. The relative fiber length of the muscles was longer in the front legs of *M. nigrocincta* (3.15 ± 0.56, *N* = 16), compared to that of *N. macrops* (2.98 ± 0.44, *N* = 16), while in the middle and the hind legs it was longer in *M. nigrocincta* (2.14 ± 0.32, *N* = 46 in the middle and 2.47 ± 0.4, *N* = 23 in the hind legs), compared to *N. macrops* (3 ± 0.44, *N* = 26 and 4.07 ± 0.37, *N* = 11 in the middle and the hind legs, respectively). Likewise, in *Odontomachus* pair, the length of muscle fibers relative to $V_{muscle}^{0.33}$ in the front leg is slightly longer in jumping *O. rixosus* (3.67 ± 0.85, *N* = 21) compared to non-jumping *O. kuroiwae* (2.73 ± 0.9, *N* = 14), while in the middle and hind leg it is more than 2 times shorter (1.45 ± 0.19, *N* = 28 and 1.73 ± 0.37, *N* = 29 in *O. rixosus* and 3.88 ± 0.82, *N* = 17 and 3.51 ± 0.46, *N* = 39 in *O. kuroiwae* middle and hind legs accordingly).

### Leg structure

#### The tibial extensor muscle in the hindlegs

**Fig. 5:**
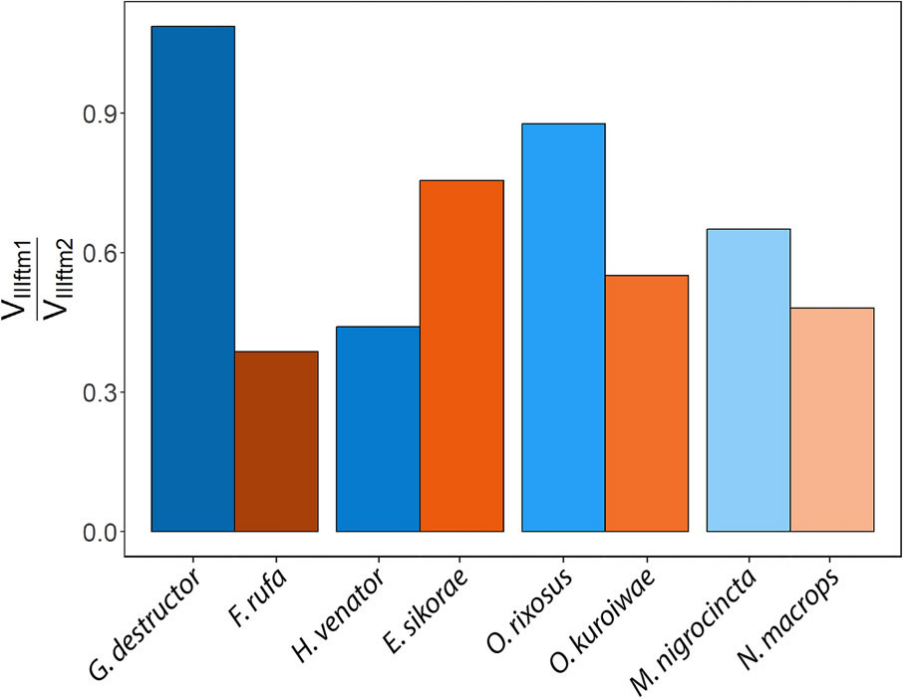
The ratio of the tibial extensor muscle to tibial flexor muscle in the hind legs. The blue bars represent jumping ants and orange bars represent non-jumping ants. The ratio of the extensor muscle to flexor muscle is larger in *G. destructor, O. rixosus*, and *M. nigrocincta* than in their non-jumping pairs, except for *H. venator.* The ratio of the tibial extensor to tibial flexor muscle is smaller than in all ants, except for *G. destructor*, where the volumes of these muscles are almost equivalent.

The pairwise comparison of the ratio of the intrinsic leg muscles that control the femoro-tibial joints (Fig. 5) shows that in *Gigantiops, Myrmecia*, and *Odontomachus*, the ratio of the extensor muscle to flexor muscle is larger than in their respective non-jumping pairs. Specifically, the ratio in the *G. destructor* and *F. rufa* pair was 1.08 and 0.38, respectively; in *O. rixosus* and *O. kuroiwae* pair 0.87 and 0.55, respectively, and in the *M. nigrocincta* and *N. macrops* pair it was 0.65 and 0.48, respectively. Thus, jumping ants have relatively larger tibia extensor muscles than non-jumping ants, except for the *H. venator* and *E. sikorae* pair. Here, the ratio of the extensor muscle to flexor muscle was 0.44 and 0.75, respectively. However, the tibial extensor muscles (ftm1) in all ants are smaller than the tibial flexor muscles (ftm2), except for *G. destructor*, where the ratio of extensor muscle to flexor muscles is 1.08, slightly larger than one.

#### The lengths of legs

In most jumping ants, forelegs are the shortest and hindlegs are the longest (Table 1 and Supplementary Table S3). This is evident in *G. destructor*, where the average length (±s.d.) of the two front legs of was 8.93 ± 0.23 mm, compared to 10.03 ± 0.13 mm and 14.7 ± 0.28 mm for the middle and hind legs, respectively (Supplementary Table S3). The ratio of the leg lengths was thus 1:1.1:1.6 (front: middle: hind) (Table 1). A trend is seen in non-jumping species; e.g., in *F. rufa*, the average length was 7.08 ± 0.53 mm, 7.24 ± 0.07 mm, and 9.03 ± 0.06 mm, respectively and the leg length ratio was 1:1:1.3. However, the hind leg length is 159% of the body length in *G. destructor* and 116% of the body length in *F. rufa*. Additionally, *G. destructor* have longer femora compared to tibiae in their hind legs, while *F. rufa* have longer tibiae compared to femora.

**Table 1:** The leg lengths in jumping and non-jumping ants

		Middle leg		Hind leg		Ratio of leg lengths			
Species	Body length (mm)	Tibia	Femur	Tibia	Femur	Hind	Middle	Front	Hind leg length (% of body length)
Gigantiops destructor*	9.24 ± 0.224	2.98 ± 0.067	3.18 ± 0.049	4.58 ± 0.072	4.78 ± 0.029	1.6	1.1	1	159
*Formica rufa*	7.79 ± 0.26	1.91 ± 0.023	1.93 ± 0.061	2.45 ± 0.046	2.41 ± 0.023	1.3	1	1	116
Harpegnathos saltator*	11.8 ± 0.624	2.08 ± 0.008	2.49 ± 0.102	2.83 ± 0.016	3.12 ± 0.033	1.4	1	1.1	92
*Euponera sikorae*	10.93 ± 0.208	1.5 ± 0.053	1.92 ± 0.072	1.86 ± 0.055	2.28 ± 0.09	1.3	1	1	83
Odontomachus rixosus*	11.07 ± 0.066	1.93 ± 0.063	2.55 ± 0.061	2.5 ± 0.085	3.15 ± 0.061	1.3	1	1	96
*Odontomachus kuroiwae*	8.45 ± 0.301	1.68 ± 0.004	2.01 ± 0.161	2.16 ± 0.061	2.66 ± 0.06	1.3	1	1.1	107
Myrmecia nigrocincta*	12.25 ± 0.19	3.01 ± 0.052	3.15 ± 0.083	4.06 ± 0.015	4.27 ± 0.039	1.5	1.1	1	122
*Nothomyrmecia macrops*	8.66 ± 0.39	1.53 ± 0.03	1.52 ± 0.016	2 ± 0.089	1.94 ± 0.05	1.4	1.1	1	83

*Note:* * indicates jumping ants.

The average length of the front legs of *H. saltator* was 8.46 ± 0.22 mm, the middle legs 7.92 ± 0.04 mm, and the hind legs 10.91 ± 0.12 mm (Supplementary Table S3), so the ratio of leg lengths was 1.1:1:1.4. Whereas for *E. sikorae*, the respective measurements were 7.19 ± 0.25 mm, 7.13 ± 0.11 mm, and 9.02 ± 0.26 mm, and the ratio was 1:1:1.3. The hind leg length is 92% of the body length in *H. saltator*, while in *E. sikorae* it is 83%.

The average length of the front, middle, and hind legs of *O. rixosus* were 8.63 ± 0.04 mm, 8.22 ± 0.06 mm, and 10.62 ± 0.24 mm, respectively, as such the ratio of leg lengths was 1:1:1.3. Meanwhile, in *O. kuroiwae* the average leg lengths were 7.46 ± 0.23 mm, 6.9 ± 0.1 mm, and 9.01 ± 0.21 mm, resulting in the leg length ratio of 1.1:1:1.3. Additionally, the hind leg length is 96% of the body length in *O. rixosus* and 107% in *O. kuroiwae*. Thus, jumping *Odontomachus* have shorter hind legs relative to the body length compared to the non-jumping *Odontomachus.*

The average length of the front, middle, and hind legs of *M. nigrocincta* were 10.15 ± 0.18 mm, 10.94 ± 0.17 mm, and 14.93 ± 0.07 mm, respectively, resulting in a leg length ratio of 1:1.1:1.5, while in *N. macrops* the average leg lengths were 5.34 ± 0.41 mm, 5.81 ± 0.09 mm, and 7.23 ± 0.08 mm, respectively, with a ratio of 1:1.1:1.4. The hind leg length is 122% of the body length in *M. nigrocincta*, while it is 83% in *N. macrops*. The non-jumping *N. macrops* has longer tibiae than femora in both middle and hind legs, while in *M. nigrocincta*, the femora are longer than the tibiae (Table 1).

### Intraspecific variation and random error check

From Supplementary Fig. S4, we can see a slight variation in the muscle volume between rep 0–1 and rep 2–3; this variation could be attributed to the ethanol concentration and length of storage time (Marquina et al. 2021; Leonard et al. 2022). However, intraspecific variation is much less compared to interspecific variation. Moreover, a random error check (Supplementary Fig. S5) demonstrates that the error during the segmentation step and computing material statistics is negligible and confirms that the variation in the muscle volumes between species cannot be attributed to measurement error.

### Jumping kinematics in *M. nigrocincta*

During a jump, *M. nigrocincta* first moves its body forward in the direction of the jump while maintaining contact with all three pairs of legs on the ground. The ant then retreats and lowers its body to the ground in preparation to take-off. During take-off, in the first six milliseconds the ant gradually raises its head. The first pair of legs leave the substrate 4–7 ms after lowering the body closest to the ground. Following this, the middle leg leaves the ground. After 4.26 ± 0.94 ms, the hind leg leaves the platform (mean ± s.e.). The entire take-off sequence lasts 19.94 ± 0.92 ms. The take-off acceleration in *M. nigrocincta* was 21.43 ± 2.49 m s^−2^ and the take-off velocity was 0.44 ± 0.03 m/s (mean ± s.e.).

## Discussion

*A priori*, the jumping ability in distantly related ant lineages could be associated with different biomechanical and morphological adaptations. However, we found consistent changes whereby trochanter depressor muscles (scm6) in the meso- and metathorax were relatively enlarged across these independent evolutions. Indeed, the first PC (Principal component) axis in the PCA analysis of the absolute muscle volumes separates jumping and non-jumping species rather than grouping them by phylogeny (Fig. 3). Below, we match the specificities of jumping behavior observed in each lineage, such as stereotypical sequence of movements of different legs or the use of the metasoma, with corresponding muscles that play a crucial role in these movements and uncover a secondary pattern in which the most relevant muscles are enlarged.

### Changes in relative muscle size

**Fig. 6:**
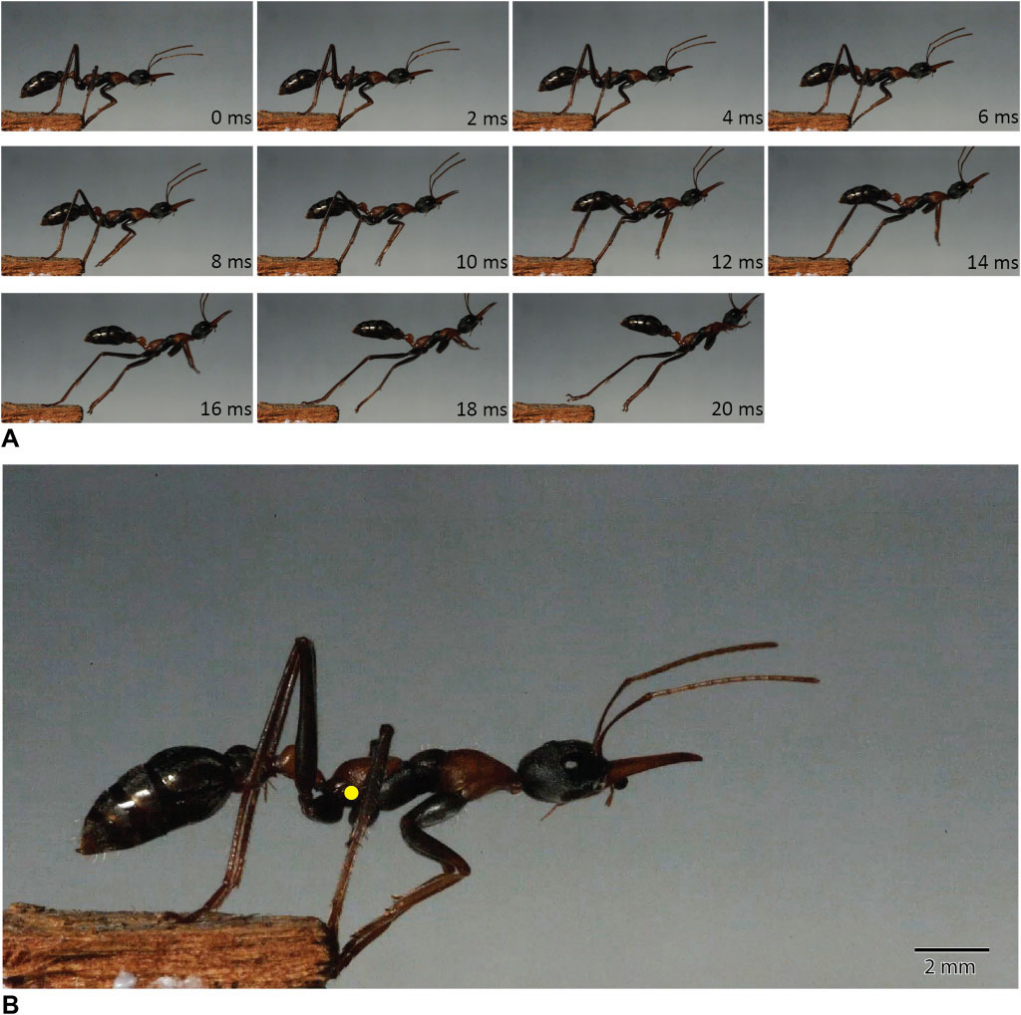
Jumping kinematics in *Myrmecia nigrocincta*. A. Sample frames at 2 ms interval illustrating the jump choreography. B. Yellow circle indicates the center of mass in *M. nigrocincta* which was tracked during the jump sequence to measure their acceleration.

Our results suggest that various muscle groups are modified in jumping ants. The depressor muscles (scm6) of both mid and hind legs are greatly enlarged in jumping ants compared to non-jumping ants. Leg depressor muscles are involved in pushing the ant body upward. Conversely, one of the coxal remotor muscles (scm2), small in the first place, are usually reduced further in jumping ants in both the middle and hind legs, except in the middle leg of *H. saltator*. It is possible that this reduction could be due to the limited space in the rigid mesosoma of ants: enlargement in the volume of one muscle may require the reduction in the volume of another (Fig. 2 and Supplementary Fig. S3). The other coxal remotor muscles (scm3), however, are enlarged in the middle legs of *H. saltator* and in both middle and hind legs of *M. nigrocincta*. In the *Harpegnathos* and *Euponera* pair, the relative volume of the mesocoxal remotor muscles are 10 times larger in the jumping ant. Moreover, these muscles extend to the anterior part of the mesonotum in *H. saltator*, which is unusual for coxal muscles. These changes may be explained by the jumping technique of this genus, which primarily uses the middle legs to jump via a fast “back-rowing” motion (Tautz et al. 1994). Previous reports suggested synchronous usage of both middle and hind legs in *M. nigrocincta* (Tautz et al. 1994). Thus, the enlargement of scm3 muscle in both middle and the hind legs might indicate that they may contribute to the jump. However, our investigation into the jumping kinematics at a higher temporal resolution revealed that in *M. nigrocincta* the middle legs lift-off slightly before the hind legs (Fig. 6, Supplementary File. 1). In a previous kinematic study by Tautz et al. (Tautz et al. 1994), the ants jumped across a horizontal gap, while in our current study, *M. nigrocincta* ants jumped to a vertical feature, necessitating body orientation for vertical landing. The use of hind legs would result in spinning the body backward, due to the COM being between the middle and the hind legs. This variation in the timing of when the mid- and hind-legs leave the platform could be based on whether they need to land on a horizontal or vertical surface. Further study on the effect of platform orientation on the jumping performance and temporal engagement of legs is needed. The increase in the relative volume of the mesial coxal remotor muscles is not substantial in other jumping ants. Lastly, *G. destructor* which rotates the metasoma while jumping, has relatively larger petiole levator muscles (IA1). These differences in muscle mass suggest that different morphological modifications are related to the observed variation in the jumping technique. It should be noted here that the jumping behavior of *Odontomachus* remains unknown. While we observed the enlargement of the trochanter depressor muscle and reduction of the posterior remotor muscle of the coxa in jumping *Odontomachus*, we cannot definitively link these adaptations to jumping without further behavioral observations. Future studies investigating the jumping behavior of *Odontomachus* would provide valuable insights into the evolution of jumping mechanisms in this ant species.

The ratio of the tibial extensor muscle to tibial flexor muscle is less than one in most ants (Fig. 5), which is a characteristic of grasping legs in other insect lineages (Földvári et al. 2019). In *G. destructor*, the ratio is 1.08, so that *G. destructor* has almost equivalent volume of the tibial extensor and flexor muscle, which is a characteristic of walking legs in other insects (Földvári et al. 2019). In *Gigantiops, Myrmecia*, and *Odontomachus*, the ratio of the tibial extensor to flexor muscle is in fact larger than that of their non-jumping counterparts. However, the fact that overall, the tibial extensor muscle is smaller than the flexor muscle suggests that ants may not rely as much on the tibial extensor muscle to jump.

If we compare the relative muscle volumes across genera, *Myrmecia* has an enlarged volume compared to the non-jumping sister lineage *Nothomyrmecia*. However, within *Myrmecia*, we see a surprising pattern: non-jumping *Myrmecia* have similar trochanter depressor muscle volumes as jumping *Myrmecia* species. It is possible that some of the species have a cryptic jumping ability. Despite their fame as “jumping jack ants,” studies on the locomotion modes across *Myrmecia* species are fragmentary and contradictory (Wheeler 1922; Snodgrass 1942; Clark 1943; Brown 1953). To better understand this phenomenon, we plan to conduct further research that combines kinematics with analysis of muscle volumes. This will help us to clarify the patterns we have observed and determine whether some *Myrmecia* species possess cryptic jumping abilities.

### Changes in muscle architecture

Above, we showed that there is an increase in size-specific muscle volume. An increase in muscle volume can be invested in area, in length, or in both. To understand whether jumping ants preferentially invest the additional muscle volume in PCSA or in fiber length, we directly compared the muscle architecture of the trochanter depressor muscles. The relative PCSA of the trochanter depressor muscles of the front (Ipcm8), the middle (IIscm6), and hind legs (IIIscm6) of jumping ants was larger compared to non-jumping counterparts, except in the trochanter depressor muscle of the front legs of *O. rixosus* and *M. nigrocincta*, where the relative PCSA was smaller. On the other hand, the normalized mean fiber length of the trochanter depressor muscle was shorter in jumping ants compared to their non-jumping counterparts in all three legs, except in the front leg of *O. rixosus* and *M. nigrocincta.* This shows that the jumping ants preferentially invest the increased volume of the trochanter depressor muscle into PCSA, except in the front legs of *O. rixosus* and *M. nigrocincta.*

There is no clear difference in the pennation angle of the trochanter depressor muscles between jumping and non-jumping ants. In *Odontomachus* pairs, the mean pennation angle in the middle and hind legs was larger in *O. kuroiwae* compared to *O. rixosus*, this change in the pennation angle is small: the force “loss” associated with pennation is $cos\ ( 8 ) = \ 0.99$ in *O. rixosus*, and $cos\ ( {27} ) = \ 0.89$ in *O. kuroiwae*. Thus, a more than three-fold difference in pennation angle only results in about 10% reduction in instantaneous force capacity.

This parallelism among jumping ants with regards to the increase in relative volume of the trochanter depressors scm6 in the mid and hind legs is particularly relevant due to the anatomical peculiarities of this muscle. Unlike the other trochanter depressor (ctm3) which originates inside the coxa (as do the trochanter levator pair, ctm1–ctm2), trochanter depressor scm6 is an extrinsic muscle which originates in the thorax (e.g., dorsal pleural areas, furcal arms, and/or notum of mesosoma) (Aibekova et al. 2022). This general anatomical arrangement already results in the muscle being longer than any of the other trochanter muscles in non-jumping ants, while in jumping ants it was possible for this muscle to enlarge into proportions that are effective for upward action, occupying a large portion of the otherwise free thoracic cavity. Moreover, being extrinsic to the coxa, the action of muscle scm6 is achieved independently of the promotion and remotion of the leg controlled by the coxal muscles. The result is that jumping ants can effectively swing the mid and hind legs backward to push their body forward, while at the same time generating the strong upward lift from the extrinsic leg depressor transmitted from the thorax to the trochanter by way of the long scm6 tendon.

### Jump mechanism—elastic recoil or direct muscle contraction?

We have identified consistent differences in the relative volume and architecture of leg muscles across jumping and putatively non-jumping ants. We now assess if observed jump performance can be explained by direct contraction of these muscles, or whether contribution from recoiling elastic elements is required. To this end, we first calculate the total work and average power requirements of observed jumps from published data and from our kinematics data presented here, $W\ = \frac{1}{2}\ m{\upsilon }^2$, and $P\ = \frac{W}{t}\ $, respectively; here, $m$ is the body mass, $\upsilon $ is the peak take-off velocity, and $t$ is the take-off time (see Table 2). We neglect gravitational potential energy in these calculations, as it makes a marginal contribution in small animals (see Scholz et al. 2006; Labonte 2023). Next, we estimate the muscle volume required to achieve this work or power output, making use of the observation that the work and average power density—the maximum amount of work and average power a unit muscle mass can generate—are relatively conserved across disparate taxa, ${W}_\rho \approx 70\ {\rm{J}}/{\rm{kg}}$, and ${P}_\rho \approx 350\ {\rm{W}}/{\rm{kg}}$ (Alexander R McNeill 2003; Biewener and Patek 2018; we assume a muscle density of $1040\ {\rm{kg}}/{{\rm{m}}}^3$). In a last step, these volume estimates may then be compared to the measured muscle volume for the trochanter depressor muscles of middle and hind legs. While it is possible that multiple muscles can be involved in powering the jump, our estimation was based on the assumption that the trochanter depressor muscles provide the primary force for upward lift; thus, we did not factor in the power output from the other leg muscles. Comparison of the power-based muscle volume assesses the possibility that jumps could be driven by direct muscle contraction; comparison of the muscle volume estimated via the work requirements, in turn, probes the possibility that the jump may have been meaningfully enhanced by recoil of elastic elements. Previous work has suggested that muscle can convert at most a third of its work density into elastic strain energy, provided that the spring constant of the involved spring-like elements takes its optimal value (Sutton et al. 2019); we thus multiply the work estimate by three, to derive a conservative lower bound for the required muscle volume. In *G. destructor, H. saltator*, and *M. nigrocincta* (see Table 2), the total muscle volume would suffice to power the jumps directly, or to drive them via elastic recoil. Indeed, Baroni Urbani et al. (1994) assumed that jumps of *H. saltator* are powered solely by muscle, based on the low take-off velocity; but they also did not exclude the possibility of energy storage. We looked for evidence for any of the three locking elements identified by Földvári et al. (2019) in the micro-CT scans of the femoro-tibial joints in all jumping species; none were found in any of the jumping ants (Supplementary Fig. S6). Similarly, bush crickets, which use direct muscle contraction to power their jumps, do not possess a locking mechanism (Földvári et al. 2019). This suggests that jumps in *G. destructor, H. saltator*, and *M. nigrocincta* may indeed be driven by direct muscle contraction alone.

**Table 2:** The parameters for the calculations of the energy requirements

Species	*G. destructor*	*H. saltator*	*M. nigrocicnta*
Jumping behavior	uses the middle and hind legs synchronously, rotates metasoma	uses the middle legs to give final propulsion	uses the middle first and next the hind leg
Body mass (kg)	0.0000155	0.000032^a^	0.0000266
Take-off speed (m/s)	0.6^b^	0.7^b^	0.44
Take-off time (s)	0.0275^b^	0.02^b^	0.0199
Work (J)	0.28 × 10^−5^	0.78 × 10^−5^	0.26 × 10^−5^
Power (W)	0.10 × 10^−3^	0.39 × 10^−3^	0.13 × 10^−3^
Muscle volume based on work requirements (m^3^)	1.15 × 10^−10^	3.23 × 10^−10^	1.06 × 10^−10^
Muscle volume based on power requirements (m^3^)	2.79 × 10^−10^	1.08 × 10^−9^	3.55 × 10^−10^
Measured total muscle volume (m^3^)	4.1 × 10^−10^	1.29 × 10^−9^	4.94 × 10^−10^

*Note:* Data are from Baroni Urbani et al. (1994)^a^ and Tautz et al (1994)^b^.

It is remarkable that the increase in relative muscle volume appears to be invested dominantly in PCSA (see also Püffel et al. 2021 for a similar result in leaf-cutter ant mandible closer muscle). The functional advantage of this preferential investment is not obvious, because both the work and the power output of muscles depend only on muscle volume. In *G. destructor, H. saltator*, and *M. nigrocincta*, the increase in peak net force arising from the increase in PCSA would result in shorter take-off times but may leave peak-speed unaffected (Labonte 2023), suggesting that it serves to enable rapid escape maneuvers. Future work is required to address this conjecture in more detail.

## Conclusion

In this study, we describe morphological adaptations associated with the evolution of forward jumping in ants. We found that all jumping ants, including *Gigantiops, Harpegnathos, Odontomachus*, and *Myrmecia*, have enlarged the relative volume of trochanter depressor muscles in the middle and hind legs, as well as a reduced relative volume of the posterior remotor muscle of the coxa. These findings indicate a common pattern of musculoskeletal modifications in jumping ants, suggesting parallel evolution of jumping mechanisms, through modifications of the leg system without latch mechanisms. However, different sets of muscles are enlarged based on which body parts are involved in jumping: medial remotor of the coxa of the middle leg in *Harpegnathos*; medial remotor of the coxa of both middle and hind legs in *Myrmecia*; levator of the petiole and extensor of the tibia in *Gigantiops*. This secondary variation suggests that while the overall pattern of morphological adaptations for jumping is shared among these ants, specific modifications may vary depending on the species and the utilization of different leg segments during the jumping motion, which could be considered an example of many-to-one mapping. To fully understand the functional significance of these muscle adaptations, further research is required to investigate the specific contributions of each muscle to the jumping techniques employed by these ants. Based on direct comparison of the observed vs. possible work and power output during jumps, we suggest that direct muscle contractions suffice to explain jumping performance, in *G. destructor, H. saltator*, and *M. nigrocincta*. These results help to elucidate morphological aspects of how forward jumping has evolved in ants and shed light on how biomechanical systems and behaviors co-evolve to generate diversity.

## Supplementary Material

obad026_Supplemental_Files

## 3D models

**Model 1.** 3D model of the skeletomuscular system of mesosoma of *Gigantiops destructor*. https://skfb.ly/oJMPD

**Model 2.** 3D model of the skeletomuscular system of mesosoma of *Harpegnathos saltator*. https://skfb.ly/oJMPH

**Model 3.** 3D model of the skeletomuscular system of mesosoma of *Euponera sikorae*. https://skfb.ly/oJMPO

**Model 4.** 3D model of the skeletomuscular system of mesosoma of *Myrmecia nigrocincta*. https://skfb.ly/oJMPv

**Model 5.** 3D model of the skeletomuscular system of mesosoma of *Nothomyrmecia macrops*. https://skfb.ly/oJMQo

**Model 6.** 3D model of the skeletomuscular system of mesosoma of *Odontomachus rixosus*. https://skfb.ly/oJMQ8

**Model 7.** 3D model of the skeletomuscular system of mesosoma of *Odontomachus kuroiwae*. https://skfb.ly/oJMQ9
