# Supplementary figures and images for: Parallel And Divergent Morphological Adaptations Underlying The Evolution of Jumping Ability in Ants
Source: Integr Org Biol. 2023 Jul 25;5(1):obad026. doi: 10.1093/iob/obad026 (PMC10401624; doi:10.1093/iob/obad026)

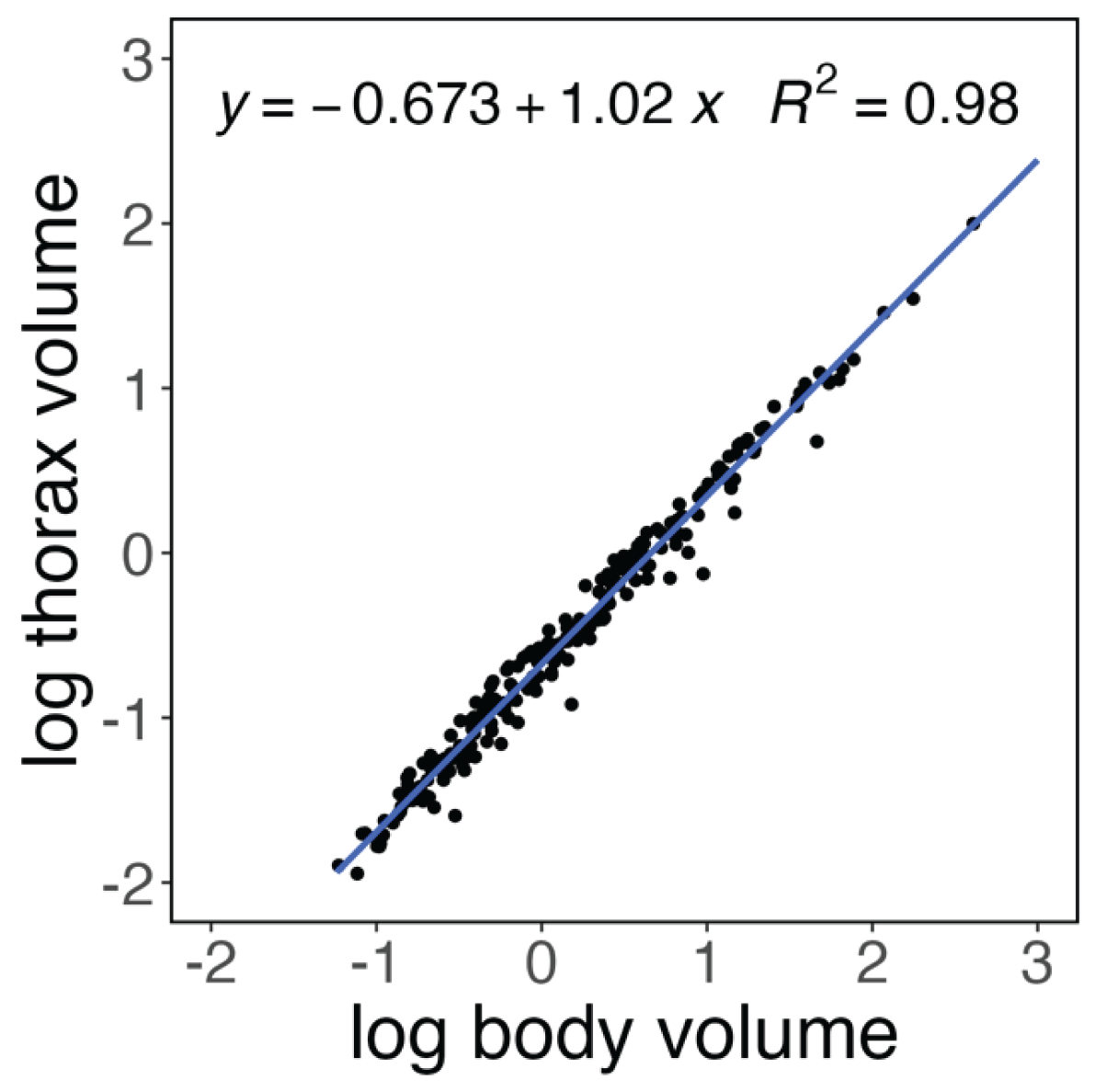

Supplement: obad026_Supplemental_Files [file obad026_supplemental_files.zip › SupplementalFigureS1.tif]

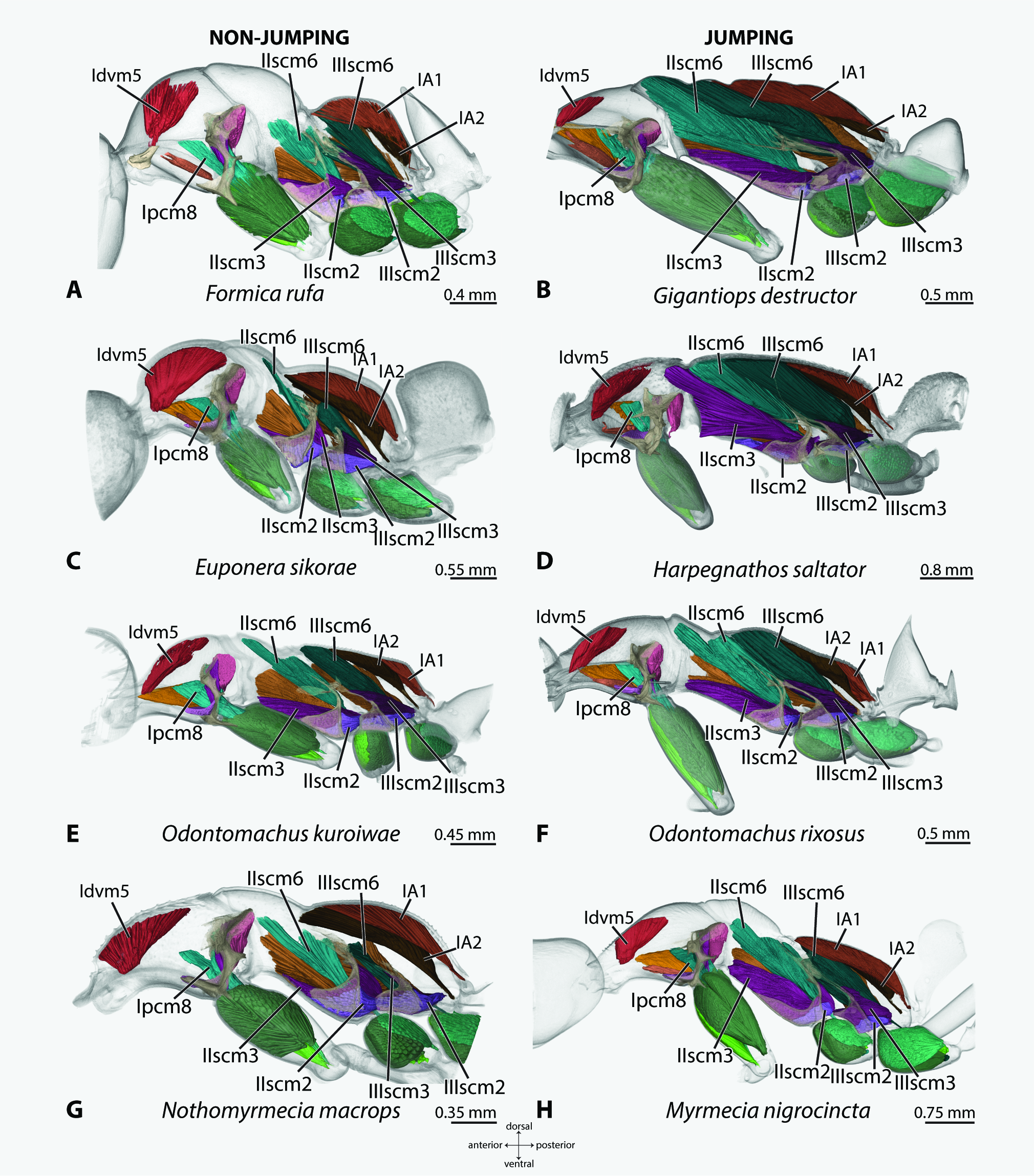

Supplement: obad026_Supplemental_Files [file obad026_supplemental_files.zip › SupplementalFigureS2.tif]

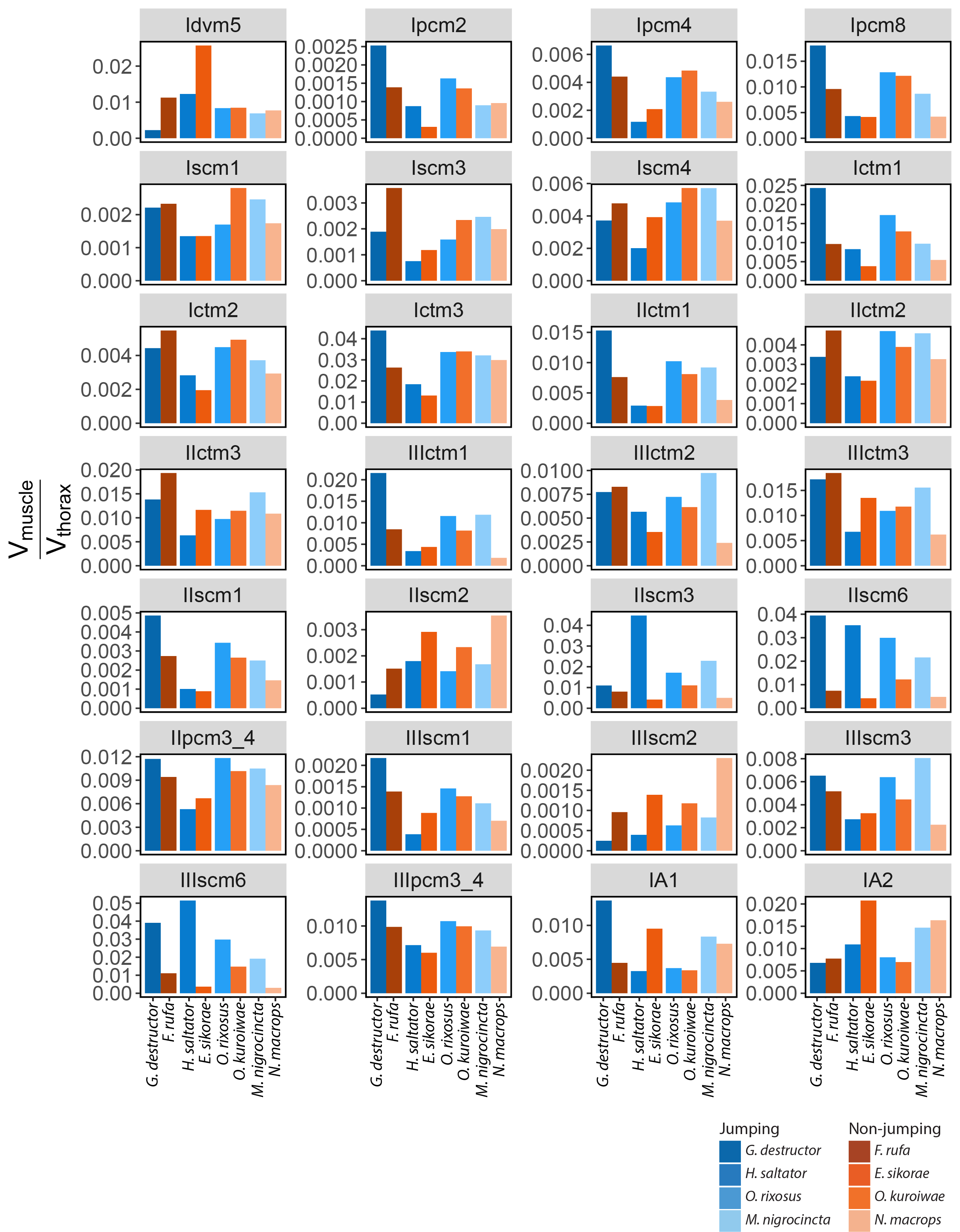

Supplement: obad026_Supplemental_Files [file obad026_supplemental_files.zip › SupplementalFigureS3.tif]

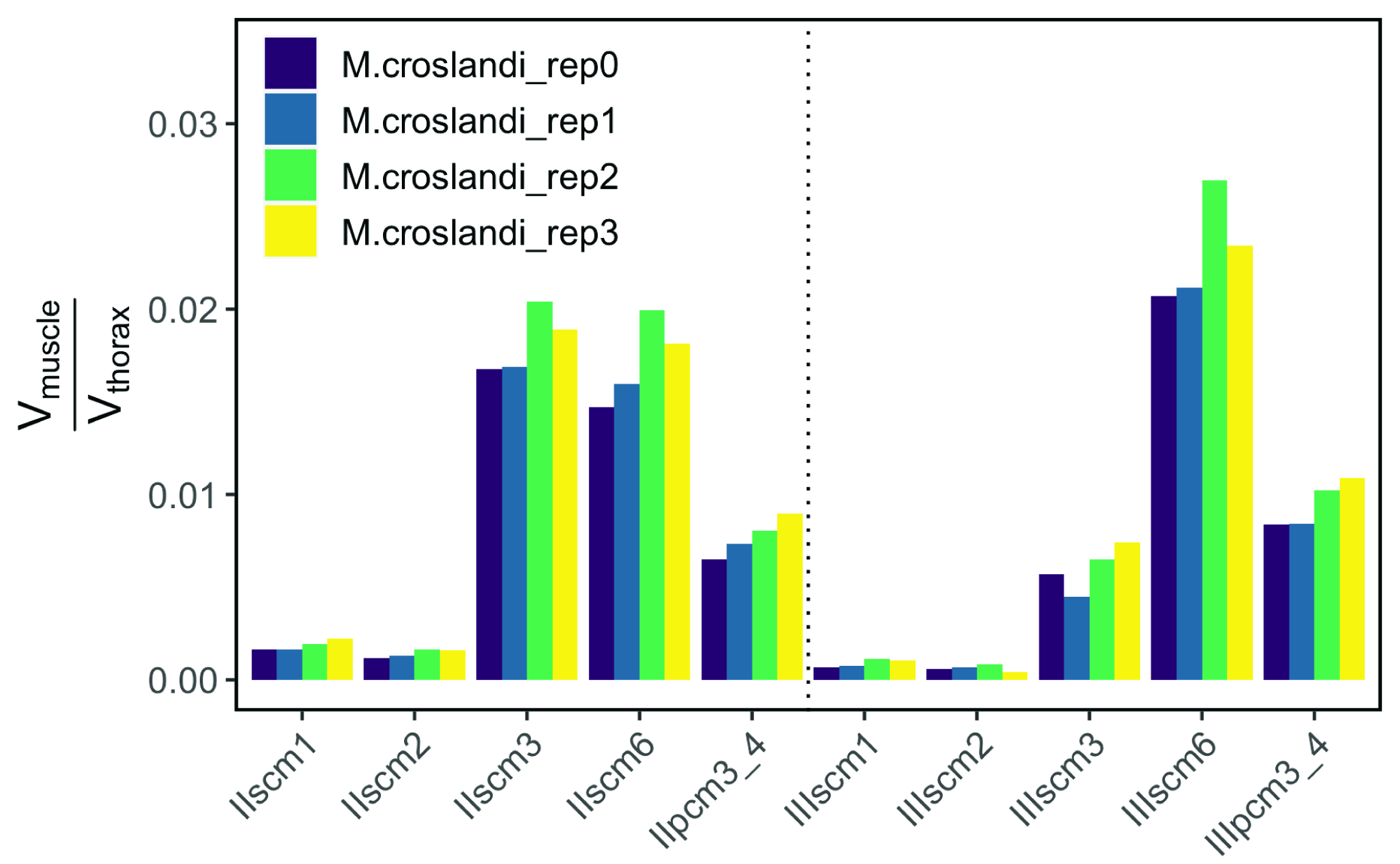

Supplement: obad026_Supplemental_Files [file obad026_supplemental_files.zip › SupplementalFigureS4.tif]

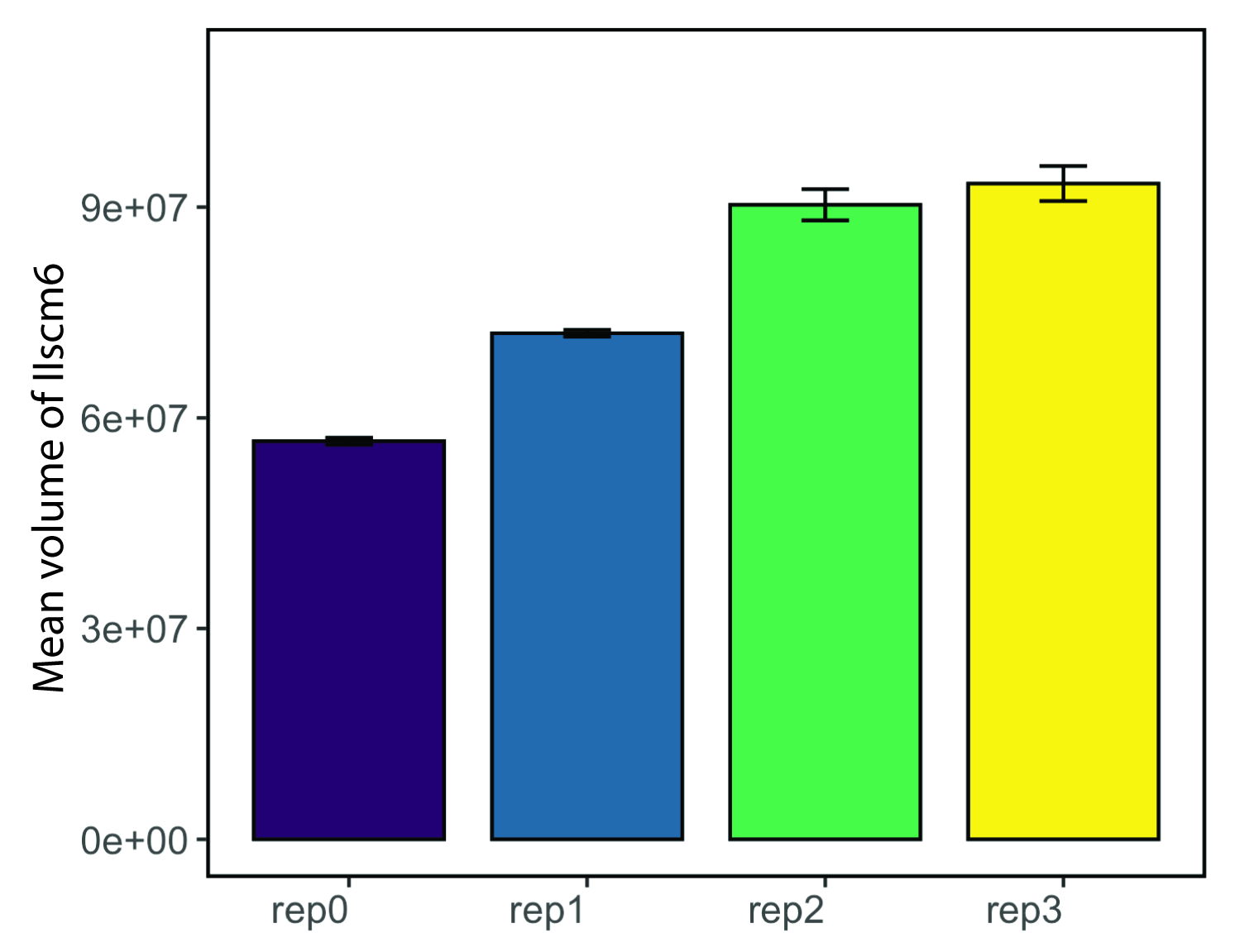

Supplement: obad026_Supplemental_Files [file obad026_supplemental_files.zip › SupplementalFigureS5.tif]

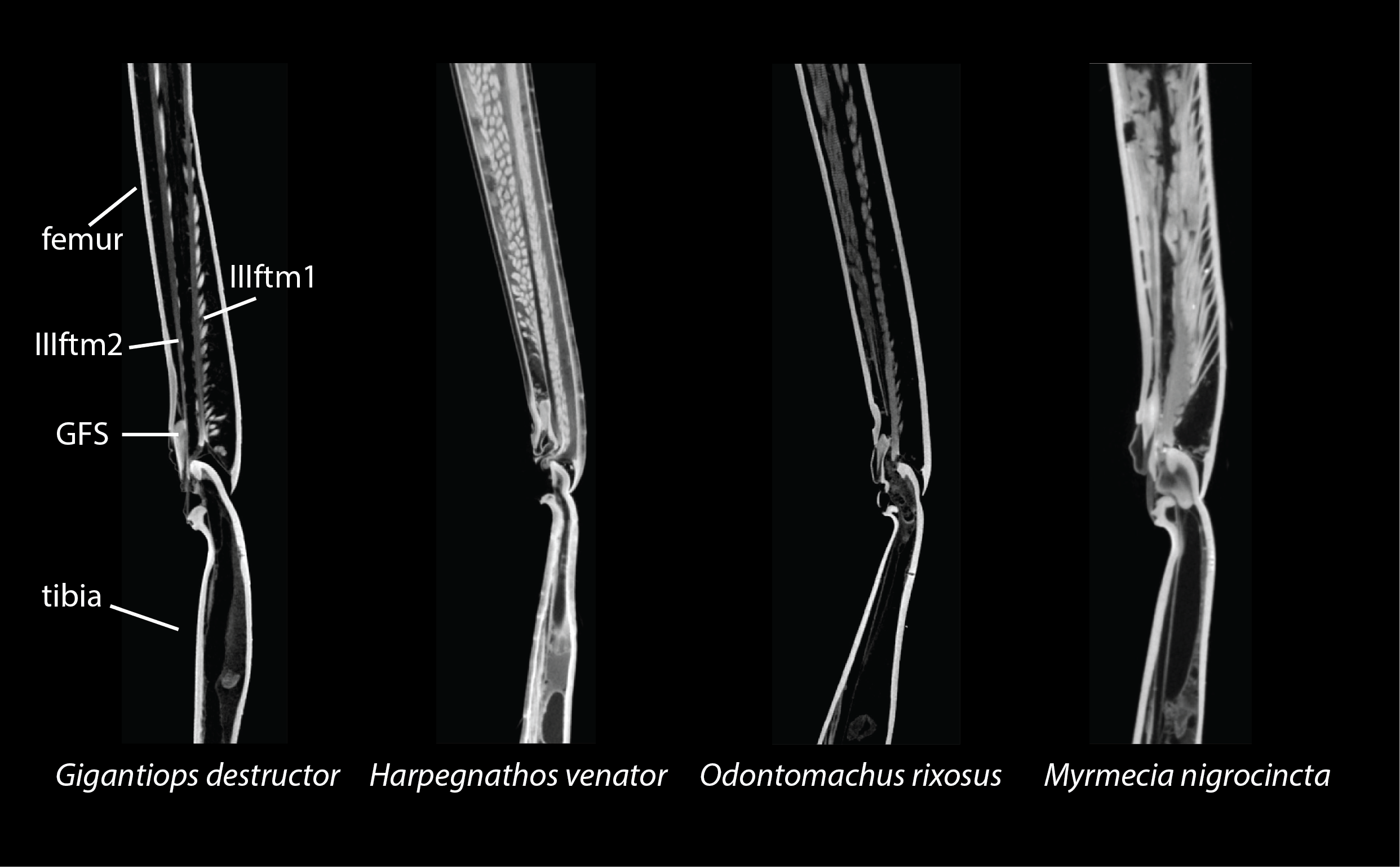

Supplement: obad026_Supplemental_Files [file obad026_supplemental_files.zip › SupplementalFigureS6.png]
